# Real-life safety of Emtricitabine/Tenofovir Alafenamide/Bictegravir

**DOI:** 10.1371/journal.pone.0289132

**Published:** 2023-08-09

**Authors:** Nicola Squillace, Elena Ricci, Paolo Maggi, Lucia Taramasso, Barbara Menzaghi, Giuseppe Vittorio De Socio, Stefania Piconi, Benedetto Maurizio Celesia, Giancarlo Orofino, Eleonora Sarchi, Giovanni Francesco Pellicanò, Filomena Simeone, Laura Valsecchi, Alessandra Bandera, Giovanni Cenderello, Letizia Attala, Goffredo Angioni, Katia Falasca, Antonio Cascio, Olivia Bargiacchi, Antonio Di Biagio, Paolo Bonfanti, for the CISAI Study Group

**Affiliations:** 1 Infectious Diseases Unit, Fondazione IRCCS San Gerardo dei Tintori, Monza, Italy; 2 Fondazione ASIA Onlus, Buccinasco (MI), Italy; 3 Infectious Diseases Unit, AORN Sant’Anna e San Sebastiano, Caserta, Italy; 4 Infectious Diseases, San Martino Hospital Genoa, University of Genoa, Genoa, Italy; 5 Unit of Infectious Diseases, ASST della Valle Olona, Busto Arsizio (VA), Italy; 6 Unit of Infectious Diseases, Santa Maria Hospital, Perugia, Italy; 7 Unit of Infectious Diseases, A. Manzoni Hospital, Lecco, Italy; 8 Unit of Infectious Diseases, Garibaldi Hospital, Catania, Italy; 9 Division I of Infectious and Tropical Diseases, ASL Città di Torino, Torino, Italy; 10 Infectious Diseases Unit, S.Antonio e Biagio e Cesare Arrigo Hospital, Alessandria, Italy; 11 Infectious Diseases, G. Martino Hospital -University of Messina, Messina, Italy; 12 1st Department of Infectious Diseases, ASST Fatebenefratelli Sacco, Milan, Italy; 13 Infectious Disease Unit, Fondazione IRCCS Ca’ Granda Ospedale Maggiore Policlinico, Milan, Italy; 14 Infectious Diseases Department, Sanremo Hospital, Sanremo, Italy; 15 SOC 1 USLCENTRO FIRENZE, Unit of Infectious Diseases, Santa Maria Annunziata Hospital, Florence, Italy; 16 Infectious Diseases Unit, SS Trinità Hospital, Cagliari, Italy; 17 Clinic of Infectious Diseases, Department of Medicine and Science of Aging, G. D’Annunzio University, Chieti-Pescara, Chieti, Italy; 18 Unit of Infectious Diseases, Department of Health Promotion, Mother and Child Care, Internal Medicine and Medical Specialties, University of Palermo, Palermo, Italy; 19 Unit of Infectious Diseases, Ospedale Maggiore della Carità, Novara, Italy; 20 Department of Medicine, University of Milano-Bicocca, Milano (MI), Italy; Walter Reed Program - Nigeria, NIGERIA

## Abstract

**Introduction:**

Integrase strand transfer inhibitors (INSTI) are one of the most prescribed drug classes for the treatment of HIV infection worldwide. Emtricitabine/Tenofovir Alafenamide/ Bictegravir (FTC/TAF/BIC) has been evaluated in randomized clinical trials; few studies have verified tolerability and safety in clinical practice. Our aim was to investigate the metabolic and hepatic safety in a real-life setting of FTC/TAF/BIC.

**Materials and methods:**

Consecutive people living with HIV infection (PLWH) enrolled in the SCOLTA project, switching to or initiating their first antiretroviral treatment with FTC/TAF/BIC were included. PLWH with HBV co-infection were excluded. Metabolic and hepatic variables were collected at T0 and T1, were defined as baseline and 6-month follow-up respectively, and their modifications were analysed using the paired t-test and the analysis of variance.

**Results:**

Five hundred and thirty-nine PLWH with at least one follow-up visit were included in the analysis. Mean age was 48 years (±12.1), 74% were male, 16.1% were naïve to antiretrovirals (ART). At T1, ART-experienced PLWH showed a significant reduction of total cholesterol (TC) and triglycerides, and a slight increase in blood glucose (BG) and ALT. On the contrary, in ART-naïve PLWH blood lipids significantly increased, although with an unaffected TC/high density lipoprotein (HDL)-c ratio, while alanine aminotransferase (ALT) decreased significantly, mainly in those with altered baseline level. The treatment interruptions were 45 (8.4%) over the whole observation period, 13 (2.4%) due to AEs. The most frequent AEs were related to the central nervous system (6 events of depression, insomnia, headache, agitation) and 3 PLWH discontinued the regimen because of grade 1–2 weight gain.

**Conclusions:**

In ART-experienced PLWH switching to FTC/TAF/BIC a significant improvement of lipid profile occurred but with significant BG and ALT variation without clinical relevance. In ART-naïve PLWH, blood lipids increased even though lipid profile did not worsen, and a trend towards normalization of liver enzymes was suggested. FTC/TAF/BIC is well tolerated in the real life setting.

## Introduction

HIV infection management has changed dramatically in the last 20 years, with a significant improvement in life expectancy and quality of life of people living with HIV (PLWH) [1].

Integrase strand transfer inhibitors (INSTI) are one of the most prescribed drug classes for the treatment of HIV infection worldwide. HIV treatment guidelines of Europe, Unites States of America and developing countries include INSTI based therapy in the favourite first line antiretroviral therapy (ART) regimens [2–4].

Randomized clinical trials, both in ART-naïve and ART-experienced PLWH [5, 6], showed that Emtricitabine/Tenofovir Alafenamide/Bictegravir (FTC/TAF/BIC) is a powerful drug combination, and data from real-life confirmed both the efficacy and its good tolerability [7, 8].

However, recent data from a large cohort demonstrated a significant weight gain and a probable increased cardiovascular risk after introduction of INSTI [9, 10]; on this issue, no data on Bictegravir exposure were available because data were censored after 2019. Obesity and overweight are increasing problems also in general population in the last years and this issue could affect PLWH independently of HIV and ART.

Pathogenesis of weight gain as adverse event (AE) of ART is still unknown, but an effect of INSTI on adipose tissue in animal and human models has been suggested [11, 12]. INSTI may promote weight gain both by inhibition of beige adipocytes and by white adipocytes hypertrophy associated with insulin resistance [11, 12].

Few data are available about AEs occurring during treatment with FTC/TAF/BIC, when used in a real-life setting. The aim of our study is to describe AEs and metabolic abnormalities in a prospective cohort of PLWH initiating FTC/TAF/BIC in a network of Italian Infectious Diseases Centres.

## Methods

We analysed data from the SCOLTA (Surveillance Cohort Long-Term Toxicity Antiretrovirals/antivirals) prospective database. The SCOLTA project, a multicentre observational cohort study, started in 2002 and prospectively follows PLWH who start treatment with new antiretroviral drugs, to identify toxicities and AEs in a real-life setting [13]. The SCOLTA project utilises an on-line pharmacovigilance program (www.cisai.it) and currently involves 30 Italian Infectious Disease Centres.

Briefly, both ART-naïve and ART-experienced PLWH can be included in SCOLTA, if they are >18 years and agreed to enter the study. Clinical data collected include sex, age, ethnicity, weight, height, CDC stage, and previous ART history at baseline. Laboratory data include HIV-RNA, CD4 +T cell count, and biochemical data, recorded at baseline and are prospectively collected in anonymous form in a central database every six months after the enrolment. AEs are collected prospectively as soon as they are clinically observed.

The first participant was enrolled in the BIC cohort in July 2019 and the enrolment is still ongoing. We selected people with at least one follow-up visit or those who interrupted before the first follow-up visit, for this analysis. We evaluated whether the metabolic profile significantly modified during the first six months of treatment with BIC, analysing the change from baseline of metabolic and hepatic variables, such as weight, total cholesterol (TC), low-density lipoprotein cholesterol (LDL-c), high-density lipoprotein cholesterol (HDL-c), TC/HDL-c ratio, triglycerides (TGL), blood glucose (BG), and TGL/HDL-c ratio. To evaluate the hepatic safety, besides the aspartate aminotransferase (AST) and alanine aminotransferase (AST) levels, we used a marker of Non-Alcoholic-Steato-Hepatitis (NASH), the AST to Creatinine Non-Alcoholic Steato-Hepatitis (ac-NASH) ratio, that was calculated according to the literature [14].

### Ethics approval and consent to participate

The SCOLTA Project was conducted in accordance with the Declaration of Helsinki. It was approved by Local Ethics committee L. Sacco Hospital, Milano, Italy, on September 18th, 2002, emended on June 13th, 2013 (protocol no. 352/2013), on December 20th, 2019 (Protocol no. 54085/2019) and on March 3rd, 2020 (Protocol no. 2020/EM/029). All patients included gave their written informed consent to participate in the study.

#### Statistical analysis

Data were described using mean and standard deviation (SD) for normally distributed continuous variables, median and interquartile range (IQR) for not normally distributed continuous variables and frequency (%) for categorical and ordinal variables.

Intragroup change from baseline (T0) to 6-month follow-up (T1) was evaluated using the paired t-test on mean (95% confidence interval, CI). Changes from baseline between the two cohorts were compared using the analysis of variance, and a multivariable general linear model including variables that were significantly different between groups, at baseline (variables reported in the footnotes).

All Ps were two-sided, at the significance level <0.05. Statistical analyses were performed using SAS for Windows 9.4 (SAS Institute, Cary, NC).

## Results

Five hundred and thirty-nine PLWH were enrolled in the BIC cohort, with a median observation time of 16 months (IQR 8–21). Mean age was 48 years (SD 12.1). Most PLWH were experienced (452, 83.9%), male (399, 74.0%) and with undetectable HIV-viral load (VL) (368/452 experienced PLWH, 81.4%). The mean body mass index (BMI) was 25.4 Kg/m^2^ (SD 4.7). Median CD4 cell count was 600 cell/mm^3^ (IQR 436–828) and 308 cell/mm^3^ (IQR 112–533) for ART-experienced and ART-naïve PLWH respectively.

**Fig 1:**
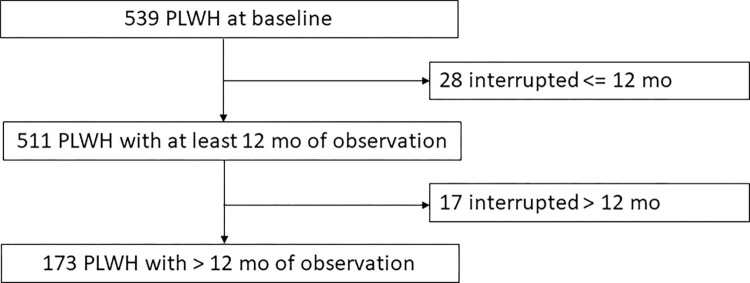
Flow chart of the treatment interruptions.

Previous ART was FTC/TAF/elvitegravir/cobicistat (EVG/COBI) in 159 PLWH (39.4%), FTC/TAF/dolutegravir (DTG) in 66 PLWH (14.6%) and other TAF-including regimens in 88 PLWH (19.5%). Only 22 PLWH were on a TDF-based regimen prior to the switch to BIC. The complete clinical characteristics of this sample are shown in Table 1 and the flow chart in Fig 1.

**Table 1:** Baseline characteristics of 539 patients on Emtricitabine/Tenofovir Alafenamide/Bictegravir, enrolled in the SCOLTA cohort.

Variables at enrolment	ART-experienced N = 452 (83.9%)		ART-naïve N = 87 (16.1%)		P*
	N or mean or median	% or SD or IQR	N or mean or median	% or SD or IQR	
**Age,** years	49.2	SD 11.7	41.5	SD 12.1	<0.0001
**Male sex**	333	73.7%	66	75.9%	0.67
**Caucasian**	398	88.0%	76	87.4%	0.86
**Risk factor for HIV acquisition**					
Sexual	282	62.4%	71	81.6%	
IDU	66	14.6%	4	4.6%	
Other/ND	104	23.0%	12	13.8%	0.002
**BMI,** Kg/m^2^ (n = 408)	25.8	SD 4.8	23.2	SD 3.5	<0.0001
**Weight,** Kg	75.6	SD 15.2	69.9	SD 12.5	<0.0001
**HCV coinfection**	82	18.1%	6	6.9%	0.003
**HIVRNA>40** copies/micrL	83	18.4%	87	100%	<0.0001
**Previous ART**					
FTC/TAF/EVG/COBI	159	39.4%	-	-	
FTC/TAF/DTG	66	14.6%	-	-	
Other DTG-based	42	7.7%	-	-	
Any other TAF-including	88	19.5%	-	-	
TDF-including (7 included in “other DTG-based”)	22	4.9%	-	-	
Other	33	7.3%	-	-	
Unknown	49	10.8%	-	-	
**CD4+,** cells/mm3	600	IQR 436–828	308	IQR 112–533	
**Total cholesterol,** mg/dL	194	SD 42	171	SD 46	<0.0001
**HDL-C,** mg/dL	54	SD 19	49	SD 19	0.004
**LDL-C,** mg/dL	111	SD 38	99	SD 38	0.008
**TGL,** mg/dL	115	IQR 85–170	97	IQR 77–149	0.04
**BG** (in 494 non-diabetic pts), mg/dL	93	SD 17	89	SD 13	0.049
**BG** (in 28 diabetic pts), mg/dL	165	SD 72	170	SD 26	0.90
**Diabetes**	24	5.3%	6	6.9%	0.55
**AST,** IU/dL (n = 390)	22	IQR 18–27	25	IQR 19–31	0.02
≥40 IU/dL	20	4.4%	12	15.6%	0.008
**ALT**, IU/dL (n = 423)	22	IQR 16–31	23	IQR 17–31	0.33
≥40 IU/dL	41	11.8%	11	14.3%	0.56
**acNASH** (n = 388)**	2.65	IQR 2.06–3.34	3.31	IQR 2.58–4.59	<0.0001
<4.15	272	87.5%	52	67.5%	
4.15–7.73	33	10.6%	21	27.3%	0.0001
≥7.74	6	1.9%	4	5.2%	

acNASH: Non-Alcoholic Steato-Hepatitis score; ALT: aspartate aminotransferase; ART: antiretroviral therapy; AST: aspartate aminotransferase; BG: Blood Glucose; BMI: Body Mass Index; COBI: cobicistat; DTG: dolutegravir; eGFR: estimated Glomerular Filtration Rate; EVG: elvitegravir; FTC: emtricitabine; HCV: Hepatitis C Virus; HDL-C: High Density Lipoprotein; IDU: Intravenous Drug User; IQR: Inter Quartile Range; LDL-C: Low Density Lipoprotein-Cholesterol; M: Male; ND: not determined; SD: Standard Deviation; TAF: tenofovir alafenamide; TDF: tenofovir disoproxil fumarate; TGL: Tryglicerides.

*Variables were compared using the analysis of variance, or the Mann-Whitney test or the chi-square test as appropriate

**Sometimes the sums do not add up to the total because of missing values.

At T1, CD4 increased by 27 cells/mm^3^ (95% CI 9, 46, p = 0.004) in ART-experienced and by 189 cells/mm^3^ (95% CI 145, 232, p<0.0001) in ART-naïve PLWH. Out of 86 ART-naïve PLWH (1 was lost to follow-up), 72.1% had T1 HIVRNA <40 copies/mL, and out of 83 ART-experienced with baseline HIVRNA>40 copies/mL, 47.6% was under this cut-off value. Among ART-experienced PLWH, 86.7% had maintained their HIV-VL < 40 copies/mL (data not shown in the Tables).

Table 2 shows the mean change from baseline, in ART-experienced and ART-naïve people.

**Table 2:** Change from baseline (T0) to 6-month follow-up (T1), by naive status.

	ART-experienced N = 452			ART-naïve N = 87			P*
	T0 Mean SD or median (IQR)	T1 Mean SD or median (IQR)	T1-T0 Mean (95% CI)	T0 Mean SD or median (IQR)	T1 Mean SD or median (IQR)	T1-T0 Mean (95% CI)	
Weight, Kg	75.6 ± 15.2	76.0 ± 13.8	0.3 (-0.2, 0.8)	69.9 ± 12.5	71.9 ± 12.3	**1.4 (0.4, 2.2)**	0.035
% change			**0.7 (0.1, 1.4)**			**2.1 (0.9–3.3)**	0.051
TC, mg/dL	194 ± 42	188 ± 40	-**5.4 (-8.6, -2.2)**	171 ± 46	183 ± 38	**15.0 (7.3, 22.6)**	<0.0001
HDL-c, mg/dL	54 ± 19	55 ± 18	0.1 (-1.0, 1.3)	48 ± 19	52 ± 17	**4.8 (1.9, 7.8)**	0.002
LDL-c, mg/dL	111 ± 38	108 ± 35	-2.5 (-5.5, 0.4)	99 ± 38	108 ± 31	**9.8 (3.4, 16.2)**	0.001
TC/HDL-c ratio	3.68 (2.91–4.62)	3.55 (2.93–4.29)	**-0.22 (-0.44, -0.01)**	3.68 (3.08–4.68)	3.70 (3.05–4.23)	-0.13 (-0.34, 0.09)	0.53
TGL, mg/dL	115 (85–170)	104 (76–153)	**-13.1 (-20.4, -5.8)**	97 (77–149)	99 (77–131)	0 (-13.5, 13.5)	0.09
TGL/HDL-c ratio	2.25 (1.65–3.64)	2.04 (1.29–3.24)	**-0.31 (-0.55, -0.07)**	2.25 (1.48–3.96)	2.06 (1.29–3.00)	-0.34 (-0.74, 0.06)	0.92
BG in non-diabetic, mg/dL	93 ± 17	96 ± 21	**2.2 (0.5, 4.0)**	89 ± 13	89 ± 12	0.4 (-2.6, 0.4)	0.29
BG in diabetic, mg/dL	165 ± 72	168 ± 70	2.3 (-30.2, 34.9)	170 ± 26	173 ± 72	32 (-108, 171)	0.53
eGFR, mL/min	86.3 ± 21.9	83.5 ± 20.5	**-2.5 (-2.9, -1.1)**	105.1 ± 29.4	89.9 ± 23.7	**-15.3 (-19.9, -10.8)**	<0.0001
AST, IU/dL	22 (18–27)	23 (18–28)	-0.6 (-3.4, 2.1)	25 (19–31)	22 (19–27)	-3.8 (-7.8, 0.3)	0.22
ALT, IU/dL	22 (16–31)	23 (16–34)	**2.4 (0.8, 4.0)**	23 (17–31)	19 (14–26)	**-5.8 (-11.0, -0.7)**	0.0002
acNASH**			-0.22 (-0.69, 0.25)			**-0.95 (-1.54, -0.35)**	0.059
<4.15	218 (86.5%)	224 (88.9%)		47 (69.1%)	60 (88.2%)		
4.15–7.73	30 (11.9%)	22 (8.7%)		17 (25.0%)	7 (10.3%)		
≥7.74	4 (1.6%)	6 (2.4%)		4 (5.9%)	1 (1.5%)		

acNASH: Non-Alcoholic Steato-Hepatitis score; ALT, aspartate aminotransferase; ART: antiretroviral therapy; AST: aspartate aminotransferase; BG: Blood Glucose; eGFR: estimated Glomerular Filtration Rate; HDL-c: High Density Lipoprotein; IQR: Inter Quartile Range; LDL-c: Low Density Lipoprotein-Cholesterol; SD: Standard Deviation; TC: Total Cholesterol; TGL: Tryglicerides.

Bold: p<0.05 for change from baseline (paired t-test)

*T1-T0 comparison by naïve status (analysis of variance)

** PLWH with both T0 and T1 values: ART-experienced n = 252, ART-naïve n = 68

As regards metabolic parameters, at T1, in ART-experienced PLWH we observed that TC varied by -5.4 mg/dl (95% CI -8.6, -2.2, p = 0.001) and TGL -13.1 mg/dl (95% CI -20.4, -5.8, p = 0.0005). The TC/HDL-c ratio significantly declined by -0.22 (95% CI -0.44, -0.01, p = 0.04). We also observed that the TGL/HDL-c ratio decreased (-0.31, 95% CI -0.55, -0.07, p = 0.01) (Ps of change from baseline are not shown in the Table).

On the other hand, blood glucose (BG) increased of 2.2 mg/dl (95% CI 0.5, 4.0, p = 0.01) in non-diabetic PLWH, and alanine aminotransferase (ALT) increased of 2.4 IU/dL (95% CI 0.8, 4.0, p = 0.004). In details, the change was 3.5 IU/dL (95% CI 2.1, 4.8, p <0.0001) in PLWH with ALT≤40 IU/dL at baseline and -7.1 IU/dL (95% CI -17.9, 3.8, p = 0.19) in those with baseline ALT>40 IU/dL. In the group of PLWH switching from a TDF-containing regimen, ALT increased by 4.8 IU/dL (95% CI -5, 14.6, p = 0.31): two people with normal baseline level had ALT>40 UI/dL at T1.

Thirty-two ART-experienced PLWH (7.1%) with ALT ≤40 IU/L at baseline had ALT >40 IU/L at T1: among them, only 1 PLWH showed a grade 2 increase (>2.5 x upper limit of normal). Nineteen experienced PLWH had persistent elevation of ALT both at baseline and at follow up.

As regards PLWH that were ART-naïve at study entry, weight, TC, LDL-c, and HDL-c showed significant increases, while ALT and acNASH decreased significantly. However, the lipid profile did not worsen as indicated by the TC/HDL-c ratio, that although not significant showed a trend towards decline (-0.13, 95% CI -0.34, 0.09, p = 0.23).

Repeating the analyses with the exclusion of PLWH on statins at baseline, we found similar results.

Among ART-naïve PLWH, ALT change from baseline was significant (-5.8, 95% CI -11.0, 0.7, p = 0.03). However, in those with ALT≤40 IU/dL it was -1.8 UI/dL (95% CI -5.0, 1.4, p = 0.26), with a marked decrease noticeable mainly in 9 PLWH with altered baseline ALT (-32.3 IU/dL, 95% CI -64.8, 0.1, p = 0.05). Only one with normal ALT baseline level showed a marked increase (from 22 to 83 IU/dL), whereas 8 with baseline ALT>40 UI/dL went back to normal at T1, and 1 did not substantially changed.

At baseline, in ART-experienced PLWH ALT was correlated with TGL (Spearman rho = 0.26, p<0.0001), TGL/HDL-c ratio (rho = 0.24, p<0.0001), and glucose (rho = 0.13, p = 0.01). Correlations were still observed at T1 between ALT and TGL (rho = 0.21, p = 0.0002), TGL/HDL ratio (rho = 0.17, p = 0.003) and BG (rho = 0.11, p = 0.049). Change from baseline, however, was only significant for the correlation between ALT and BG (Pearson r = 0.14, p = 0.02).

In absolute terms, weight increased in naïve patients, but not in experienced ones (+1.4 kg, 95% CI 0.4, 2.2, vs +0.3, 95% CI -0.2, 0.8, p = 0.035).

Including in a multivariable model potential confounders such as age, sex, use of lipid lowering drugs, HCV coinfection, the associations did not significantly change.

The treatment interruptions were 45 (8.4%) over the whole observation period, 13 (2.4%) due to AEs (See Table 3).

**Table 3:** Adverse events and treatment interruptions.

Reason for treatment interruption	ART-experienced N = 452	ART-naïve N = 87
First year	21 (4.6%)	7 (8.0%)
Overall observation time	32 (7.1%)	13 (14.9%)
Treatment failure	1	
Death*	1	
Pregnancy	1	
Simplification	5	2
Other**	4	4
Lost to follow-up	8	6
Adverse events	12	1
**Grade 1–2 events causing treatment interruption**		
Musculoskeletal+CNS	2	
CNS (agitation, depression)	2	
Dermatological (itching)	1	
Weight gain	2^#^	1^##^
**Grade 3–4 adverse events causing treatment interruption**		
Increased creatinine	1	
CNS (headache, insomnia)	2	
Enteritis	1	
Osteopenia	1	
**Grade 3–4 adverse events not causing treatment interruption**		
Anaemia^§^	1	
Angina pectoris	1	
Bacterial endocarditis	1	
Diabetes	1	
Herpes Zoster		1
Increased triglycerides	1	
Pneumococcal pneumonia^§^	1	
Thrombocytopenia	1	

* Sepsis

** Patient relocated to another centre (5), drug-drug interaction (3)

^#^ +4 and +8 Kg

^##^ +4 Kg

The most frequent AEs were related to the central nervous system (6 events of depression, insomnia, headache, agitation) and 3 PLWH discontinued the regimen because of grade 1–2 weight gain (+4 Kg in an ART-naïve 45-years old man and in an ART-experienced 58-years old woman, +8 Kg after 13 months in an ART-experienced 51-years old woman). Seven PLWH (5 ART-experienced, 2 ART-naïve) modified treatment by switching to a dual therapy. During the first year of observation, 28 (5.2%) PLWH interrupted treatment, 21 (4.6%) ART-experienced and 7 (8.0%) ART-naïve.

## Discussion

In our cohort of PLWH starting FTC/TAF/BIC in a network of Italian Infectious Diseases Centres, during the first year of treatment we observed a low prevalence of adverse events, both in the ART-experienced and in the ART-naïve group such as in other cohorts [7, 8, 15]. In details a Spanish cohort of 1299 PLWH [7] with a similar proportion of ART-naïve and experienced PLWH and observation time, reported a higher global rate of treatment interruption compared to our cohort (13% vs 8.4%). Evaluating only patients with documented discontinuation (excluding PLWH lost to follow up), we observed a similar rate of treatment interruptions (5.7% in our cohort vs 5.3% in Ambrosioni et al. [7]). In other real-life studies, discontinuation rates due to AE ranged from 0 to 2.2% [8, 15].

In our population, CNS AEs were the most represented but were lower than in the other studies: 6 PLWH interrupted treatment for this reason (1.1% of the entire cohort, over 2.4% of interruptions due to AEs). In RCTs, the percentages of PLWH that reported CNS AE were higher (range 6–28.3%), although they did not lead to discontinuation [5, 6].

In our cohort only 3 PLWH interrupted treatment for excess of weight gain, but the percentage weight gain was significant in both groups and higher in ART-naïve PLWH (with borderline significance), as reported in other studies [16]. In the recent analysis of the RESPOND cohort, weight gain was not directly related to FTC/TAF/BIC, because data were censored at 2019 and BIC use was not included [9]. In real-life cohorts on FTC/TAF/BIC treatment, weight gain was not always observed; in the study of Rolle et al. [8], weight gain was not significant, and in the study of Lazzaro et al. [15] weight and BMI increase was demonstrated, but within normal ranges. The effect of FTC/TAF with INSTI on weight gain could be additive, as shown in recent analyses [9, 17].

Lipid profile improved in ART-experienced PLWH who switched to FTC/TAF/BIC, as described in other real-life experience. In details, our analyses confirmed the significant reduction of TC, as in two other studies [8, 15], and the decrease in TGL as in the study of Rolle et al. [8].

On the contrary, we did not observe a reduction in LDL-c, probably because of the differences in the previous regimen in our cohort (mostly FTC/TAF/EVG/COBI). This may be due to the different previous regimens used in other cohorts. In fact, they were predominantly protease inhibitor-based in Lazzaro et al [15], and included ritonavir or COBI in 54% of cohort in Rolle et al. [8].

We demonstrated a minimal but statistically significant increase in BG and ALT levels. The trajectories of ALT during treatment with FTC/TAF and INSTI were previously analysed [18, 19], consistently showing a trend towards the reduction of ALT, after switching from FTC/tenofovir disoproxil fumarate (FTC/TDF) to FTC/TAF.

No long term published data on hepatic safety and glucose variation of FTC/TAF/BIC are available up to now and cited study on FTC/TAF/BIC in real life setting [7, 8, 15] and RCTs [5, 6] did not reported any significant changes in ALT/AST. Recent unpublished data from ICONA cohort on 2911 PLWH switching from TDF to TAF reported an initial significant decrease of liver enzymes (at ≤ 1 year by the switch) followed by a slight increase over a median follow up of 42 months [20].

In our previous study, a significant increase in BG was observed [19] especially in the switch from FTC/TDF/EVG/COBI to FTC/TAF/BIC. In the current analysis we confirm the significant but mild increase of blood glucose as suggested by the in vivo studies on metabolic derangements of adipose tissue [11, 12].

In ART-experienced PLWH referring to our network, we found an association between baseline ALT and TGL, BG, and TGL/HDL-c ratio as a marker of insulin resistance [21]. A significant correlation was also observed between ALT and BG increase at T1. As Insulin Resistance during INSTI treatment has been demonstrated in vitro and in vivo [11, 12], our findings could justify the hypothesis that a metabolic derangement might cause an increase in ALT.

Non-alcoholic fatty liver disease is associated with Insulin Resistance and ALT increase [22] and may evolve in NASH. A non-invasive test, named the acNASH index, that combines serum creatinine and aspartate aminotransferase levels, was developed as a screening tool to identify NASH among adults [14]. For this reason, we explored the trend of the acNASH score, that in ART-naïve PLWH enrolled in our study decreased significantly, as well as ALT, suggesting a beneficial role of FTC/TAF/BIC in reducing the probably HIV-induced liver damage.

This study has some limitations. First, the Infectious Diseases Clinics involved in the SCOLTA study participated in our observational study on a volunteer basis, thus they are not formally representative of the Italian Clinic, because they were not randomly selected among Clinics at the national level. Second, the study participants were PLWH in need of initiating a new ART drug, in the Infectious Diseases Clinics participating into the SCOLTA study, and as such, not fully representative of all PLWH followed up in the participating centers. Lastly, our PLWH are aged 48 years on average, thus our results cannot be generalised to all Italian PLWH, especially those older than 55 years. The hypothesis of the role of NASH could not be confirmed because neither liver ultrasound nor fibroscan was available. acNASH is not universally used as a marker of NASH; a very different series of biological and instrumental markers have been purposed for NASH diagnosis and studies are ongoing to define the better one. However, liver biopsy remains the gold standard for the diagnosis, and it is not available for our population. We used only indirect markers on Insulin Resistance such as TRG/HDL ratio that are not widely used in clinical practice. Despite these limitations, one strength of this study is to describe a large, real-life cohort of PLWH on FTC/TAF/BIC-based regimens, followed up prospectively in a research network (SCOLTA) specifically designed to improve post-marketing surveillance of adverse reactions to ART, and with a specific expertise in AE monitoring.

In conclusion, FTC/TAF/BIC is a very well tolerated therapy with a low rate of discontinuation, mainly due to CNS events that remained, however, infrequent. The main finding was the improvement of lipid profile in PLWH with prior ART treatment. In the same group, ALT and BG increase were statistically significant, with a current limited impact on strong clinical endopoints. Our findings should be confirmed on a longer observation period, but they invite to look at metabolic and liver abnormalities during treatment with FTC/TAF/BIC.
